# High Similarity Image Recognition and Classification Algorithm Based on Convolutional Neural Network

**DOI:** 10.1155/2022/2836486

**Published:** 2022-04-12

**Authors:** Zhizhe Liu, Luo Sun, Qian Zhang

**Affiliations:** ^1^School of Art and Design, Wuhan University of Technology, Wuhan 430070, China; ^2^Glasgow School of Art, Glasgow Scotland G4 9LE, UK; ^3^School of Art and Design, Wuhan University of Technology, Wuhan 430070, China

## Abstract

Nowadays, the information processing capabilities and resource storage capabilities of computers have been greatly improved, which also provides support for the neural network technology. Convolutional neural networks have good characterization capabilities in computer vision tasks, such as image recognition technology. Aiming at the problem of high similarity image recognition and classification in a specific field, this paper proposes a high similarity image recognition and classification algorithm fused with convolutional neural networks. First, we extract the image texture features, train different types, and different resolution image sets and determine the optimal texture different parameter values. Second, we decompose the image into subimages according to the texture difference, extract the energy features of each subimage, and perform classification. Then, the input image feature vector is converted into a one-dimensional vector through the alternating 5-layer convolution and 3-layer pooling of convolutional neural networks. On this basis, different sizes of convolution kernels are used to extract different convolutions of the image features, and then use convolution to achieve the feature fusion of different dimensional convolutions. Finally, through the increase in the number of training and the increase in the amount of data, the network parameters are continuously optimized to improve the classification accuracy in the training set and in the test set. The actual accuracy of the weights is verified, and the convolutional neural network model with the highest classification accuracy is obtained. In the experiment, two image data sets of gems and apples are selected as the experimental data to classify and identify gems and determine the origin of apples. The experimental results show that the average identification accuracy of the algorithm is more than 90%.

## 1. Introduction

In recent years, with continuous research on deep learning, many problems in daily life can be solved by artificial intelligence. One of the characteristics of deep learning is that programmers no longer need to continue to write programs to complete the program as in the past. They only need to build a neural network in advance, imitate human brain thinking with artificial means, and match a sufficient amount of data to simulate the training machine itself. Then, they let the machine obey the laws of the data, and thus, it can learn from the data. At this stage, the artificial intelligence industry has become very common, and we can see the shadow of artificial intelligence in all walks of life. With the help of neural networks, the artificial intelligence industry can create brand-new technologies that liberate the human brain and complete a variety of tasks on its own. In summary, artificial intelligence can be defined as the study of human intellectual activities; that is, with the help of hardware and software, the computer can simulate this activity, so that the computer can complete the work that the human brain could only do in the past.

Generally speaking, the network structure of deep learning models is more complex and the number of parameters is often relatively large. Therefore, in order to make the model better performance, people generally rely on the corresponding manually labeled data to do the corresponding training; otherwise, there may be overfitting problems or training difficulties. However, generally in actual situations, because it is difficult to obtain enough data, data in some application fields are still seriously missing. For example, in the world, the amount of rare ores is small, and the corresponding data are often difficult to collect, and it is relatively difficult for people to collect some relevant data on endangered precious animals or plants. In addition, even if sufficient data are available, the cost of manual labeling during this period is often high. Therefore, it is difficult for people to use enough data to train the corresponding neural network model enough, which will seriously affect its performance, which may lead to more serious overfitting problems of the model. Therefore, using less data to maximize the performance of the model is a hot spot in the field of deep learning. Another issue worthy of attention in this field is that even though deep neural networks have completed simulation training based on a large number of data samples, they still cannot use the simulation results in other fields [1–7].

Image recognition technology is a very core direction, such as national security, public security, transportation, finance, industrial production lines, and food inspection. Traditional image recognition algorithms first need to preprocess the image, including image segmentation, image enhancement, and binarization refinement, and then perform manual feature extraction to establish a recognition model for corresponding features, such as Gaussian mixture model, support vector machine, hidden Markov model, and so on, and then judge the match. The extraction method has limited ability to represent the middle-level structure and high-level semantic information of the image, and traditional algorithms are time-consuming, and it is difficult to apply them in actual production [8–13].

Before 2012, the error rate of image recognition has been high (about 26%). In the ILSVRC-2012 competition, the algorithm using the convolutional neural network has achieved very good results. Since then, convolutional neural networks have become the mainstream algorithm in this field. Almost all teams have used convolutional neural networks more or less. The best results that can be achieved by image recognition are algorithms related to convolutional networks. This reflects the good performance of convolutional neural networks in computer vision from the side. Soon, in some related fields, such as face recognition and handwritten font recognition, convolutional neural networks have also achieved world-leading results. Therefore, its application fields are very wide, such as gesture recognition, face recognition, iris recognition, and vehicle recognition [14–21].

At the same time, there is almost no need to perform some preprocessing or feature value extraction on the image, which is an advantage that some other machine learning algorithms do not have. The convolution kernel of the convolutional neural layer is larger, and the number of layers in the overall structure is slightly less, so the advantages of the algorithm are not fully utilized [22–27].

Because the convolutional neural network needs a lot of data for simulation training in the initial stage, and the hardware equipment requirements of the computer itself are relatively high, it is often difficult to obtain a network with relatively good performance through training. However, in recent years, with the continuous advancement of GPUs and corresponding labeled data, convolutional neural networks have shown better and better results in dealing with image recognition or image classification problems. It is precise because of this advantage of convolutional neural networks that it is widely used in face recognition, object recognition, and other fields. In recent years, the successful application of convolutional neural networks in image recognition has received widespread attention. Generally speaking, common image recognition methods can generally be divided into the following three types: decision theory recognition, syntactic pattern recognition, and fuzzy pattern recognition. Among them, a major feature of syntactic pattern recognition is the use of several structural features to form a single recognized object, which can accurately describe the characteristics of the image. An image can usually be thought of as consisting of lines, curves, polylines, etc. Therefore, it is often combined with the knowledge of statistical decision making in mathematical statistics to reconstruct the secondary space to achieve the purpose of image recognition. Commonly used methods include similar judgment method, similar analysis method, and function classification method. Among them, fuzzy pattern recognition is mainly through training to imitate the process of identifying things in the human brain, which can not only obtain more accurate classification of objective things, but also improve the simplicity of the recognition module. Therefore, it is also a method for the above two recognition methods.

LeNet is a more traditional convolutional neural network with a model depth of only 5 layers. Although the current convolutional neural network technology can be competent for some data sets, the categories of these data sets are very few. Therefore, they are more favor to use other training data sets to train the model, such as ImageNet, so as to realize the trained convolutional neural network model, so that it has a stronger depth and breadth. With the further development of neural networks, scholars often achieve this by increasing the number of layers of the model. However, this method will cause the model parameters to increase greatly. Therefore, in order to solve such problems, GoogLeNet adopts the initial module. Its main principle is to control the cumbersome degree of calculation of model training, and it ingeniously overcomes the problem of gradient failure in reverse transfer. Muhammad has proposed another kind of deep learning model for image classification named as VGGNet [28]. After comparative analysis, the performance difference between convolutional neural network and network depth is studied systematically. Wu proposed the ResNet model for visual recognition [29]. He believes that the difficulty of training network parameters increases as the depth of the network increases. Once the model depth index exceeds a certain value, the problem of gradient explosion and gradient disappearance will occur, and the degree is positively correlated with the network depth, but the accuracy rate is reduced. Existing convolutional neural network models constantly change the depth and width of the model and, at the same time, continuously optimize the model architecture, so that its performance is significantly improved. In the image recognition task, as the current best-performing convolutional neural network, its classification performance on multiple image data sets (e.g., the ImageNet data set) has been compared with human capabilities. Therefore, on this basis, Deng, Koresh, and Ai proposed a capsule network [30–32]. It uses neuron vectors to replace the single neuron node commonly used in traditional convolutional neural networks.

## 2. Convolutional Neural Network

In this section, the convolutional neural network proposed in this paper will be introduced and discussed. In addition, its specialty compared with a conventional one is also pointed out.

### 2.1. Convolutional Neural Network Structure

The neural network refers to a multilayer network system built with a large number of neurons, and the activation layer is used to give the model a strong nonlinear fitting ability. The model will learn to extract image features and perform result mapping based on the image input by the designer and the desired result. A large amount of work can be completed through deep learning, and the designer only needs to complete the more difficult feature extraction work. The designer needs to set up the network results to improve the efficiency and accuracy of automatic extraction. The core of the neural network is based on the combination of matrix multiplication and nonlinearity. After a large number of filtering cores, the main features that can help the mapping result are effectively screened out, and useless features are removed, and then, deep learning and classification are performed on this basis. At present, the commonly used structures in engineering include inception, VGG, and ResNet. In general, the designer will use the original model to train it on the corresponding data set and, on this basis, choose the model with the ideal training result to do further improve. The model used in the project should have the characteristics of fast speed and high accuracy. The general way to reduce the model is to reduce the number of ResNet modules or the number of convolutions. Currently, the most widely used models mainly include network models such as YOLO, SSD, Mask-RCNN, and FRCNN, all of which have the advantages of high accuracy and speed. Compared with traditional recognition methods, adding a deep learning algorithm module in the image processing field has the following advantages: the algorithm itself can complete the analysis of big data independently and extract features from it, without the need for manpower to complete this tedious task.

The neural network is a model obtained by highly abstracting the operation model of the human nervous system. The basis of its structure is neurons. In the definition of biology, the components of neurons include cell bodies and processes. Processes can be further divided into dendrites, axons, and synapses. If the current passing through the neuron exceeds a certain threshold, the neuron will be activated.

Convolutional neural networks reduce the number of parameters inside the network through special connections between adjacent layers. In CNN, the latter convolutional layer can extract the data features of the previous convolutional layer and extract the higher-level feature information from the low-level feature information. The basic components of CNN are introduced below.

**Figure 1:**
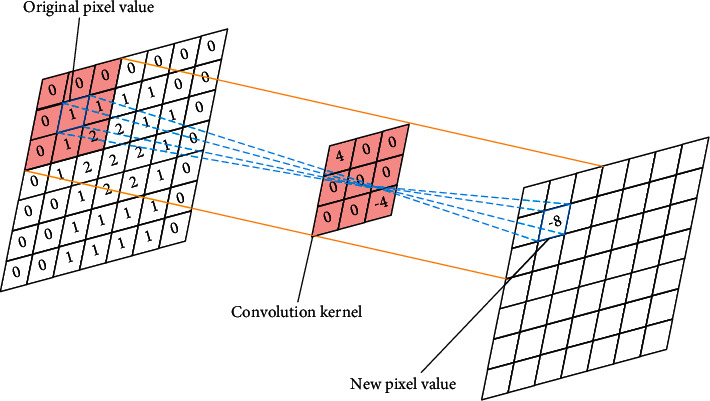
The diagram of convolution operation.

Generally speaking, in the convolutional neural layer, the larger the convolution kernel, the better the “abstraction” effect on the image, but the more parameters that need to be trained; the smaller the convolution kernel, the more finely the image can be processed, but more layers to achieve the same “abstract” effect. However, a smaller convolution kernel means more ReLU layers, which means that the entire structure is more discriminative. The convolutional layer is one of the most important components in CNN, and its main function is to use the filter (convolution kernel) to obtain the data feature information of the previous layer. The schematic diagram of the convolution operation is shown in Figure 1. The leftmost part of the figure is the original data value, the matrix of which is 7∗7, the middle part is the filter, the matrix of which is 3∗3, and the right is the output data. The value of its size is 7∗7 matrix. The filter in the figure is equivalent to a sliding window, which uses a fixed step size to regularly slide on the original data, and the mapped area is valued by the corresponding mathematical operation rules. The sliding step length set in the figure is 1, the filter slides, and the corresponding calculation is performed after each data value is moved. The figure adopts the mathematical operation rule of multiplying the filter and the corresponding data in the mapped area and then adding. Therefore, the size of the output data after the operation is the same as the size of the original data. Generally speaking, the convolutional layer has multiple filters, and the area where the filters are mapped to the input data is called the receptive field. The blue part of the input image in the figure is the receptive field. The larger the filter used, the larger the corresponding receptive field. In summary, when performing a convolution operation, the filter is equivalent to a sliding window, and each element in the filter has a corresponding weight and offset. The process of corresponding convolution operation on the mapped data can be expressed as

(1) $$\begin{array}{c} y_{n}\left(x,y\right)=\sum_{k=1}^{k_{n-1}}\sum_{m=1}^{w}\sum_{z=1}^{h}\left[y_{n-1}^{k}\left(si+m,sj+z\right)W_{n}^{k}\left(m,z\right)\right]+b_{n},\\ L_{n}\left(w\right)\frac{L_{n-1}\left(w\right)+2p-w}{s}+1,\\ L_{n}\left(w\right)=\frac{L_{n-1}\left(h\right)+2p-h}{s}+1, \end{array}$$

where yn(i,j) is the data value of the coordinate (i,j) in the output data of the n-1th convolutional layer; yn-1(i,j) is the data value of the n-1th convolutional layer in the input data, the coordinate is the data value of (i,j); *n* is the number of convolution layers; *k* is the number of channels; w is the width of the convolution kernel; *h* is the height of the convolution kernel; *s* is the convolution step length; bn is the bias parameter of the nth convolutional layer; and *p* is the number of padding.

**Figure 2:**
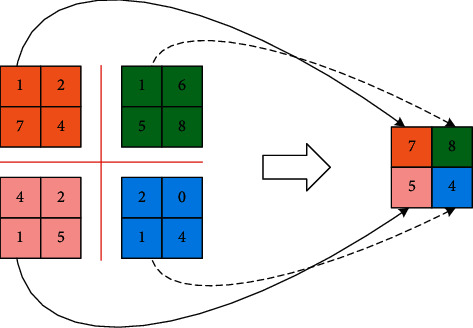
The diagram of max-pooling.

Figure 2 is a schematic diagram of maximum pooling. The left side of the figure is an input data matrix with a size of 4∗4, and the filter used is a matrix with a size of 2∗2. Assuming that the moving step length is 2, the filter performs corresponding operations after every two data values moved, so the output data are a matrix of size 2∗2. In the maximum pooling operation, the data area mapped by the filter only retains its maximum value, which effectively reduces the parameters of the input data. In order to improve the performance and robustness of the algorithm, it is necessary to subsampling the image here. If the divided areas do not overlap each other, such an algorithm is called nonoverlapping pooling; otherwise, it is called overlapping pooling. There are also two methods for calculating the output for each area: averaging (generally called sum pooling or Avg pooling) or taking the maximum value (Max pooling). This neural layer is relatively simple and does not require training. In addition, this algorithm sometimes ignores the edge part of the input image, which is also acceptable for the algorithm as a whole. If the input image is 13∗13 and the size of the area selected by the Pooling layer is 3∗3, the bottom and rightmost edge of 1 pixel will be ignored. In the classic network structure, the overlapping Max pooling algorithm that can be overlapped is used, because it can slightly reduce overfitting. A similar algorithm is also used in this article.

The activation functions used by the activation layer include ReLU, Leaky ReLU, Tanh, and Sigmoid. The above activation function is described in detail below. Among them, the expression of the ReLU function is as follows:

(2) $$\begin{array}{c} f\left(x_{r}\right)=\left\{\begin{array}{c} x_{r},x_{r}>0,\\ 0,x_{r}\leq 0, \end{array}\right) \end{array}$$

where xr is the input data. The activation layer of this function only needs to determine whether the input data value is greater than 0. If the value is less than 0, the gradient of the layer is returned to zero. If the value is greater than 0, the gradient of the layer remains unchanged. This feature effectively reduces the generation of network training, and problems are overfitting.

The expression of the Sigmoid function is as follows:

(3) $$\begin{array}{c} f\left(x_{s}\right)=\frac{1}{1+e^{-x_{s}}}, \end{array}$$

where xs is the input data. It can be seen that the Sigmoid function is a logarithmic linear model and is monotonic, so its output range is limited and it is easier to perform derivative operations.

The expression of the Tanh function is as follows:

(4) $$\begin{array}{c} f\left(x_{t}\right)=\frac{2}{1+e^{-2x_{t}}}-1, \end{array}$$

where xt is the input data. It can be seen from the above formula that the Tanh function also belongs to the log-linear model.

The expression of Leaky ReLU function is as follows:

(5) $$\begin{array}{c} f\left(x_{l}\right)=\left\{\begin{array}{c} x_{l},x_{l}\geq 0,\\ \alpha x_{l},x_{l}<0, \end{array}\right) \end{array}$$

where *a* is a constant parameter with a value range of 0∼1, and xl is the input data. The technical core of the algorithm is the weight update. This part affects the final result of image data classification. In order to get a higher classification accuracy, this article conducted many experiments, focusing on the weight update part of the convolutional layer and the pooling layer of the neural network. The feature map of the previous layer is convolved with a convolution kernel that needs to be learned to obtain a new feature map, as shown in the following formula:

(6) $$\begin{array}{c} x_{j}^{l}=f\left(\sum_{i\in M_{j}}x_{i}^{l-1}\times k_{ij}^{l}+b_{j}^{l}\right), \end{array}$$

where Mj represents the selected input feature map combination, *k* is the convolution kernel used for the connection between the i-th feature map of the input of the network layer of the first layer and the j-th feature map of the output, and *b* is the first layer of the network layer. The bias corresponding to the j-th feature map, *f,* is the activation function.

The sensitivity calculation method of convolutional neural network neurons is shown in the following formula:

(7) $$\begin{array}{c} \delta_{j}^{l}=\beta_{j}^{l+1}\text{up}\left(\delta_{j}^{l=1}\right)f^{\prime }\left(u^{\prime }\right), \end{array}$$

where up() represents the *δ* upsampling operation, *β* is the weight parameter of the j-th node of the l+1-th layer network, *δ* is the error term of the j-th node of the l-th layer network, and f′ is the full connection obtained by using gradient descent. The convergence direction and size of the layer output are influenced by u, which is the input of the fully connected layer of the neural network.

The error cost function is as follows:

(8) $$\begin{array}{c} \frac{\partial E}{\partial k_{i,j}^{l}}=\sum_{u,v}{\left(\delta_{j}^{l}\right)}_{u,v}{\left(p_{i}^{l-1}\right)}_{u,v}, \end{array}$$

where *u* and v are the two-dimensional scale coordinates of the image mapped by the convolutional layer, and *p* represents the value of the element-wise multiplication of *x* and *k* during convolution. In the down-sampling layer, the new feature map can be obtained by the following formula:

(9) $$\begin{array}{c} x_{j}^{l}=f\left[\beta_{j}^{l}\text{down}\left(x_{j}^{l-1}\right)+b_{j}^{l}\right], \end{array}$$

where down() represents the down-sampling operation. The sensitivity calculation method is as follows:

(10) $$\begin{array}{c} \delta_{j}^{l}=f^{\prime }\left(u_{j}^{l}\right)\text{conv}2\left(\delta_{j}^{l+1},\text{rot}180{\left(k_{j}^{l+1}\right)}^{\prime },{\text{full}}^{\prime }\right), \end{array}$$

where conv2 is the convolutional layer of the neural network, rot180 represents the inversion of the neuron sensitivity value and transforms the rows and columns of the feature matrix, and full' represents the output value of the image pixel of the fully connected layer, and *u* is the first layer of the network. *j* is the fully connected layer input of nodes. Finally, the gradient is calculated to obtain better network weight parameters

(11) $$\begin{array}{c} \frac{\partial E}{\partial b_{j}}{\sum_{u,v}\left(\delta_{j}^{l}\right)}_{u,v}. \end{array}$$

After updating the weights of the convolutional layer and sampling layer of the neural network, it is necessary to set detailed optimization parameters for other layers. The parameters of each layer of the convolutional neural network used in this paper are shown in Table 1. In fact, only the data in this table have been trained. The network includes 1 softmax classification layer, 2 local response normalized LRN (local response normal) layers and two random sampling layers. Many tests have found that the accuracy of image classification under this parameter is high. Among them, the principle of the LRN layer is to use the biomimetic nervous system inhibition mechanism to make the value of the larger function response becomes relatively larger and improves the generalization ability of the model. In addition, it should be noted that the model is trained with the different gem and apple positions.

## 3. Experimental Design and Analysis

The experiment was carried out on Tianhe-2, using a single node, dual CPUs, 24 computing cores, 64 GB of memory, namely, CPU E5-2692v2 24^∗^2.20 GHz. The algorithm is implemented using *Python*3.7 and Matlab (2016b). For the two data sets of gems and apples, the algorithm of this paper is used to identify gem classifications and identify the origin of apples. Among them, the gem and apple data sets each contain 20,000 images, the training set is 15,000, and the test set is 5,000.

**Figure 3:**
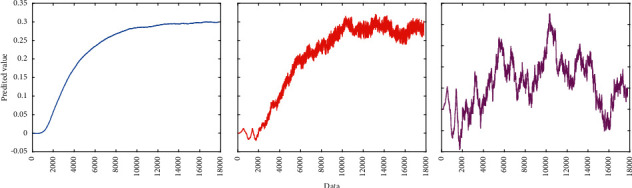
Predicted value.

The content of the input layer defined in this paper is a feature vector extracted by wavelet transform that retains image texture features. The vector is transformed from an image matrix. The original image data with the background removed can be decomposed into images through wavelet transform. Then extract the features of each subpicture and normalize it as the input of the convolutional neural network for further processing. The neural network layer constructs a fully connected pool, which includes 4096 neurons; each neuron is connected to the input layer; and each neuron receives 4096 inputs. During the training process, the loss function value is recorded and output to monitor the training progress. Set the number of cycles to 201, and when the number of cycles meets a multiple of 10, the loss value is output. The predicted is shown in Figure 3.

**Figure 4:**
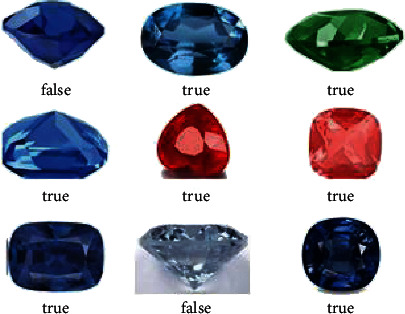
An example of preliminary classification of gem data set.

In the network structure training process, from the input layer to the softmax layer, each layer contains the parameters to be solved. Therefore, the data with category labels are used as the input, a forward propagation is performed first, the classification error is calculated, and then, the stochastic gradient descent method is used. Backpropagation is used to solve the optimal parameters so as to minimize the classification error. Part of the classification results are shown in Figure 4. Among them, the label true means the classification is correct, and the label false means the classification is wrong. The total number of classified images is initially 1,000, the number of gems is 500, and the number of training is 500. Statistics show that the classification accuracy rate is 78.55%.

**Figure 5:**
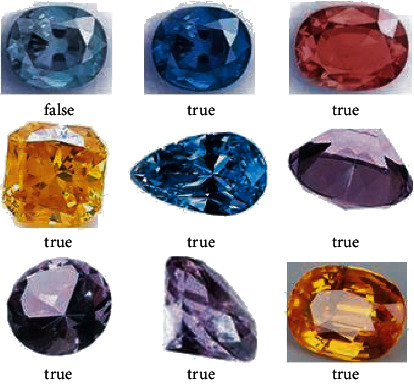
An example of classification results after CNN model optimization.

**Figure 6:**
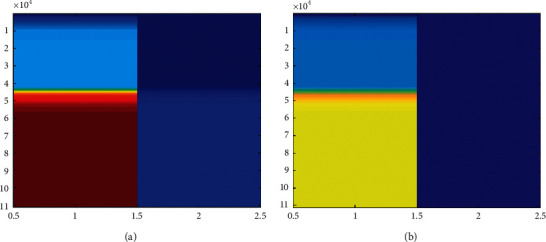
Evaluated data.

When the convolutional neural network model is in the local convergence of the gradient descent, the classification will be invalid; that is, the parameter values cannot be saved during the training process, and the weight parameters of the network model need to be re-randomized during each training process and multiple trainings. The set does not play a role in the optimization of the neural network model, and the model has poor applicability. In order to save all the parameter values in the training process, the parameter values of the historical training are directly read during the test of the same kind of data set. The experiment adds two convolutional layers to form a convolutional neural network and adds twice between the input layer and the fully connected layer: convolution and pooling. Taking the gem data set as an example, some of the classification results randomly selected after 500 trainings are shown in Figure 5. In order to improve the accuracy, and after 500 times of training, the algorithm accuracy rate reached 86.77%, which shows a better effect we get compared with Figure 4. The evaluated data are shown in Figure 6.

**Figure 7:**
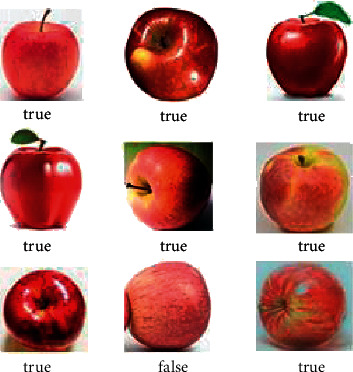
Apple data set classification results.

**Figure 8:**
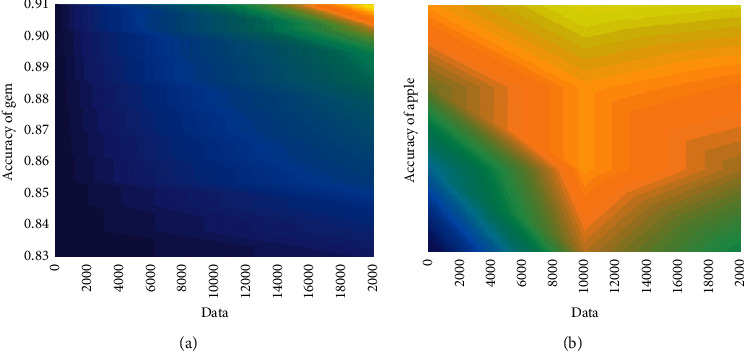
Effect of data volume on algorithm accuracy.

**Figure 9:**
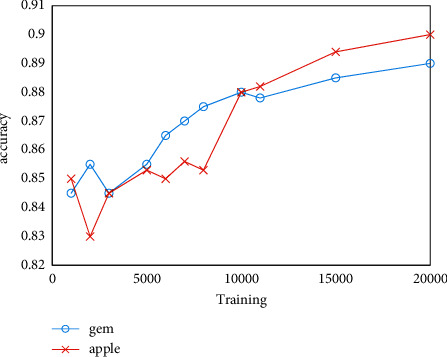
Effect of training frequency on algorithm accuracy.

In order to prove the accuracy of the algorithm in the field of high similarity image recognition and classification, an optimized algorithm was selected to test the apple data to determine its origin. The number of Apple data sets is 1,000, and the number of training is 1,000. Part of the classification results are shown in Figure 7. Statistics on the classification results show that the classification accuracy rate is 87.54%. In order to test the change law of the accuracy of the algorithm with the size of the training set data, the two data sets of gem and apple were tested, respectively, and the unit increment of the data set was set to 2000. The test results are shown in Figures 8 and 9. It can be seen from Figure 8 that as the amount of data increases, the parameters of the neural network will be continuously optimized, thereby effectively improving the accuracy of the algorithm. It can be seen from Figure 9 that under the premise that the data set remains unchanged, as the number of training increases, and the parameter values of the neural network will not fall into a local minimization, which improves the global optimization effect and accuracy.

**Figure 10:**
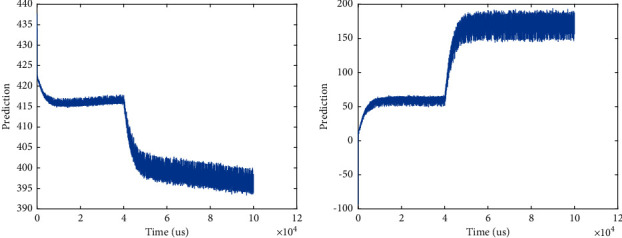
The prediction.

In order to verify the superiority of the algorithm in this paper, compare it with the classification algorithms that use wavelet transform alone and neural network alone. It can be seen that when the number of training times of the convolutional neural network algorithm is small (less than 10,000 times), the accuracy of the algorithm after many experiments is high. When the number of training times reaches 2,000 times, the accuracy rate reaches the highest 90.06%. The image classification algorithm fused with wavelet transform and convolutional neural network has higher classification accuracy. The prediction is shown in Figure 10.

## 4. Conclusion

Nowadays, the information processing capabilities and resource storage capabilities of computers have been greatly improved, which also provides support for the deepening of neural network technology. Convolutional neural networks have good characterization capabilities in computer vision tasks, such as image recognition technology. Aiming at the problem of high similarity image recognition and classification in a specific field, this paper proposes a high similarity image recognition and classification algorithm fused with convolutional neural networks. First, extract the image texture features, train different types and different resolution image sets, and determine the optimal texture difference parameter value; second, decompose the image into subimages according to the texture difference, extract the energy features of each subimage, and perform classification one-dimensional processing; then, the input image feature vector is converted into a one-dimensional vector through the alternating 5-layer convolution and 3-layer pooling of the convolutional neural network; on this basis, different sizes of convolution kernels are used to extract different convolutions of the image dimensional features, and then use unit convolution to achieve the feature fusion of different dimensional convolutions; finally, through the increase in the number of training and the increase in the amount of data, the network parameters are continuously optimized to improve the classification accuracy in the training set and in the test set. The actual accuracy of the weights is verified, and the convolutional neural network model with the highest classification accuracy is obtained. In the experiment, two image data sets of gems and apples were selected as the experimental data to classify and identify gems and determine the origin of apples. The experimental results show that the average identification accuracy of the algorithm is more than 90%.

## Figures and Tables

**Table 1:** Structure and parameters of CNN layers.

Convolutional neural network layers	Specific parameters of each layer
Cov1	96, LRN, pool32, str2
Cov2	256^∗^52, LRN, pool32
Cov3	384^∗^32
Cov4	384^∗^32
Cov5	256^∗^52, pool32, str2
Full6	4096
Full7	4096
Full8	1000 softmax

## Data Availability

The data used to support the findings of this study are available from the corresponding author upon request.
